# Prevalence of dyslalias in 8 to 16 year-old students with anterior open bite in the municipality of Envigado, Colombia

**DOI:** 10.1186/s12903-015-0063-1

**Published:** 2015-07-10

**Authors:** Andrea Ocampo-Parra, Bibiana Escobar-Toro, Valentina Sierra-Alzate, Zulma Vanessa Rueda, María Clara Lema

**Affiliations:** Faculty of Dentistry. Universidad Cooperativa de Colombia, Carrera 47 # 37 sur 18, Envigado, Antioquia Colombia; Universidad Cooperativa de Colombia, Medellin, Colombia; Epidemiology. Faculty of Dentistry. Universidad Cooperativa de Colombia, Medellin, Colombia

**Keywords:** Open bite, Speech, Language, Hearing sciences

## Abstract

**Background:**

Anterior open bite AOB is the most common malocclusion associated with speech disorders and the literature has shown that problems of occlusion involve all oral functions. AOB not only produce aesthetic and occlusal problems for the patient and modifies the union of the lips, tongue, teeth, palate, palatal rugae and oropharynx, and thus affecting the ability to communicate well with their surroundings.

The prevalence of AOB in children and adolescent in our population is unknown. Furthermore, the most frequent type of dyslalias in children with this malocclusion is also unknown. Therefore, the aim of the study was to describe the frequency and types of dyslalia in students between 8-16 years with AOB, as well as the difference in the types of dyslalia according to the magnitude of AOB.

**Methods:**

A cross-sectional study was conducted. Clinical assessment of AOB in students from the municipality of Envigado, Colombia, was performed. Students from 8 to16 years of age were examined during the second semester of 2011 and first semester of 2012. Phonoaudiological assessment was carried out in students in the mixed or permanent dentition. Exclusion criteria included children with history of systemic disease, altered skeletal development, neurological and psychiatric disorders, and residents in other departments. In addition, students undergoing orthodontic treatment at the time of evaluation or with history of previous orthodontic treatment, as well as those who did not cooperate with the oral cavity evaluation, were excluded.

**Results:**

Six thousand one hundred sixty five children were evaluated. One hundred sixty six presented AOB (prevalence: 2.7 %; 95 % CI: 2.28–3.10). Thirty four students were excluded. 26.5 % of the sample presented mild AOB, 66.7 % moderate, and 6.8 % severe. Some type of dyslalia was found in 77.4 % of the students, being distortion (75.8 %) the most common. The most frequently altered phonemes were: / d / t / s / ch / ñ /. No significant association between different types of dyslalia and AOB severity (*p*-value = 0.974) was found.

**Conclusion:**

Prevalence of AOB in Envigado is low (2.7 %). Phonation alterations are very common in children with AOB (77.8 %), and distortion is the most frequent type of dyslalia (75.8 %). In order to diagnose and treat occlusal and phonetic problems, and to avoid possible recurrence, interdisciplinary approach is recommended.

## Background

As published in the literature, occlusion problems affect all oral functions [1]. For orthodontists, anterior open bite (AOB) has been a challenge in terms of treatment and frequent recurrence [1]. Patients with AOB develop functional adaptations to swallow, masticate, breath, and speak [2]. AOB not only produces aesthetic and occlusal problems to patients, but also results in impaired mastication and articulation of certain phonemes [3] since the union of the lips, tongue, teeth, palate, palatal rugae, and oropharynx plays a significant role in the articulation of sounds to construct language [4]. Therefore, their ability to communicate properly may be compromised [2].

80 % of speech movements are carried out at the front of the mouth. Therefore, a relationship between oral defects and incorrect phonation was observed by Kimball *et al.* in 1937 (cited by Doshi *et al.* [4]).

AOB is the most common malocclusion associated with speech disorders [5]. Fimbo (cited in a literature review by Johnson *et al.* [6]) evaluated 410 patients with AOB. This study found that 63 % of children with open bite showed speech disorders. Bernstein, also cited by Johnson *et al.* [6], examined 437 children with speech disorders and found that malocclusion is not usually related to phonation problems, except in the case of AOB, where a strong relation was established. Mehnert [7] found that AOB was the most common malocclusion associated to mispronunciation of phonemes.

Oral language is affected when an abnormal function of any of the components involved in the articulation of phonemes (organ of respiration, phonation organs, and/or joint bodies) is present. In general, respiratory disorders may cause some types of stuttering, laryngeal abnormalities may cause hoarse or false voices, and alterations of articulation organs may produce dyslalias.

The concept of dyslalia corresponds to a disorder in the articulation of phonemes, either by altering or omitting some specific sounds, or by replacing sounds incorrectly. It is an inability to properly pronounce or form certain phonemes or groups of phonemes. Based on its causes, dyslalias may be classified as evolutionary, functional, audiogenic, and organic [8].

Functional dyslalias may be classified as S*ubstitution dyslalias*, which define an error in speech articulation where a sound is replaced by another; *Omission* dyslalias occur when the proper articulation of the phoneme is unknown and is not replaced by another phoneme; *Insertion dyslalias* refer to the replacement of a sound by another sound that does not correspond to the specific word; *Distortion dyslalias* occur when a phoneme is articulated incorrectly but is not replaced by a specific phoneme, or the articulation is similar, not exact, to the correct form. This is usually the result of a defective position of the articulation organs [8].

Alvarez *et al.* [5] found that patients with open bite exhibited mostly phonemes substitution followed by omission and distortion. The investigation by Pomerantz and Zeller, cited by Johnson *et al.* [6], specifically described the phonemes involved and concluded that an AOB or an edge-to-edge bite relation is significantly associated with pronunciation disorders of the phonemes s / z / th (d) / l.

Prevalence of AOB in children and adolescents from Envigado, Colombia, and the most frequent type of dyslalias in children with this malocclusion are unknown. Therefore, the aim of this study was twofold: first, to describe the frequency and types of dyslalia in students from Envigado, Colombia, between 8–16 years of age who presented AOB. Second, to establish whether a relation between types of dyslalia and AOB magnitude occurs.

## Methods

### Type of study: cross-sectional

Population: Students from the municipality of Envigado, Colombia, between 8–16 year of age, enrolled in public schools during the second semester of 2011 and the first semester of 2012. Inclusion criterion included children and adolescents with AOB in mixed dentition with fully erupted maxillary and mandibular incisors or in permanent dentition. Signing of an informed consent by the child and legal guardian to participate in the study was required. Exclusion criteria included presence of systemic diseases that cause alterations of normal skeletal development and children with neurological and psychiatric disorders. Presence of orthodontic treatment or history of previous orthodontic treatment, as well as lack of cooperation with the oral evaluation, resulted in exclusion from the study. Students who did not reside in the department of Antioquia were not considered for evaluation. This study was approved by the Ethics Committee from Universidad Cooperativa de Colombia.

Techniques and procedures: At baseline, prior authorization was obtained from the Secretary of Education to visit local elementary schools. Subsequently, a standardization of data collection instruments and a pilot test to evaluate the entire operating process were completed. Children from seven educational institutions were evaluated. Students were visually assessed by two of the researchers in order to identify those who presented AOB. Next, parents or legal guardians of children with AOB were invited to participate and signed an informed consent in the presence of two witnesses. Once approval was obtained, each patient was clinically inspected and overbite was recorded using a Boley gauge.

AOB, as defined by Bishara, is a vertical gap between maxillary and mandibular incisal edges while posterior teeth are in contact [9]. AOB magnitude was classified as low (up to 1 mm), moderate (1 to 5 mm), and severe (>5 mm) according to Dawson (1974), cited by Iwasa *et al.* [10], considering the degree of amplitude between incisors.

For speech evaluation, VSA designed and adapted a personal evaluation of Spanish spoken in Colombia to avoid misinterpretations or incorrect data analysis. This evaluation was based on the scores provided by Tobias Corredera Sánchez [11], who describes phonemes, and by Bernal and Baquero [12], who describe consonant sounds. Evaluation of articulation points and modes was as follows: articulation place: bilabial: / m / p / b /; labiodentals: / f // v /; interdental: / none /; dental: / t / d /; alveolar: / s / n / l / r / rr /; palatal: / y / ll / ch / ñ /; velar: / k / g / j / x /. Articulation mode: Occlusive: / p / b / t / d / k /; fricatives: / f //v// s / y / ll / g / j /; affricates: / ch / x /; nasal: / m / n / ñ /; lateral: / l /; vibrant: / r / rr /.

Speech assessment of each student according to pronunciation of these phonemes was classified as normal, distortion by tongue interposition, distortion by tongue thrust, substitution, or omission. Additionally, articulation organs were assessed by performing the following activities: descent of the lingual apex to the lower lip, movement of tongue tip to reach the lip corners, elevation of the lingual apex to the upper lip, lips in a kiss position, uni and bilateral cheek inflation, right and left movement of lips together, and sweeping the palate with the tongue. As a regular protocol in speech evaluation, these praxias were evaluated for coordination, skill, and symmetry of lingual and labial muscles. Furthermore, palate shape, sensitivity, and rugae thickness (normal, thick or thin) were evaluated.

Palatal rugae are located in the most anterior area of the hard palate. These rugae are anatomical references for the tongue in resting position and play a role during stomatognathic functions. The following definitions were used to describe palatal rugae: tenous palatal rugae, where a mild roughness, corresponding to pressure exerted by the tongue in resting position, was observed; pronounced palatal rugae, where hypertrophic rugae due to lack of stimulation by the tongue during resting position or swallowing were observed [13].

Palatal sensitivity must be considered since both soft and hard palate are covered by oral mucosa with high numbers of sensory receptors that provide proprioceptive information. Therefore, some aspects, such as appropriate language patterns of the tongue at rest to specify the articulation point, especially for sounds that require contact of various parts of the tongue with the palate, and swallowing function, both in the oral and initial pharyngeal phases [13], must be evaluated. Palatal sensitivity evaluation consists of carefully applying a soft sensory stimulus in the anterior-posterior direction, which may be linear or circular, and monitoring closely the reaction displayed by the subject to avoid excessive responses, like nausea. The stimulus may be applied at the palatine raphe, or at the palate edges. Parameters to determine sensitivity are: normosensitivity, hypersensitivity, and hyposensitivity [13].

Sample size: considering that the school population between 8–16 years from the municipality of Envigado for June 2011 was 22.995 and that the prevalence of AOB reported in the literature was 2 % [14], a sample size of 460 students with ABO was determined as reference for the present investigation. Based on this population and with a 50 % expected proportion of dyslalias, a 95 % confidence level, and a sampling error of 7 %, the calculated sample size of students with AOB was 132.

Analysis Plan: before the analysis, quality control of the database was performed by confirming the information included in the data collection form of 10 % of all the children who presented AOB. The absolute and relative frequencies to describe AOB in school children and its magnitude, dyslalia and types, gender, and grade were reported. Age was described as median with interquartile range (IQR), as it did not follow normal distribution.

To establish whether there was a difference between the magnitude of AOB and the type of dyslalia, Chi square test of independence was carried out. A *p* value <0.05 was considered significant. Data analysis was performed using the statistical package SPSS version 18.0 (SPSS Inc., Chicago, IL, USA).

## Results

Six thousand one hundred sixty five patients were considered and 166 subjects were identified as presenting AOB (prevalence = 2.7 %; 95 % CI: 2.28–3.10). Based on exclusion criteria, 34 patients were excluded as follows: 14 due to orthodontic treatment at the time of evaluation, nine did not show for the speech evaluation, seven refused to participate in the study, two presented neurological and psychiatric disorders, and two did not reside in Antioquia. A total of 132 patients with AOB were assessed. The median age was 11 years (IQR: 9–14); 58.3 % were female and 41.7 % male.

According to severity, 26.5 % showed mild, 66.7 % moderate, and 6.8 % severe AOB. 100 % of the individuals were capable of performing the following activities: descent of the lingual apex to the lower lip, movement of tongue tip to reach the corners of the lips, attainment of kiss position, and bilateral cheek inflation. One child (0.8 %) did not attain unilateral cheek inflation. 97 % performed praxias elevating the lingual apex to the upper lip, 99.2 % swept the palate with the tongue, and 89.4 % moved their lips together to right and left (Table 1).

**Table 1:** Characteristics of 132 school children with Anterior Open Bite (AOB) between 8–16 year from Envigado, Colombia

Variable	*n* (%)
AOB magnitude	
Mild	35 (26.5)
Moderate	88 (66.7)
Severe	9 (6.8)
Upper lip in contact with lingual apex	128 (97)
Unilateral cheek inflation	131 (99.2)
Movement of lips together to right and left	118 (89.4)
Type of dyslalia	
Distortion	100 (75.8)
Substitution	1 (0.8)
Replacement and distortion	1 (0.8)
No dyslalia	30 (22.7)

Palate shape was typical in 32 % of children, high and narrow in 59 %, and low in 8.3 %. Palatal rugae were normal in 63.6 %, thick in 24.2 %, and thin in 12.1 %. Palatal sensitivity was normal in 71.2 % of patients, reduced in 25 %, and increased in 3.8 % (Table 1). No association was found between palatal sensitivity and palatal rugae (*p* value = 0.089).

In the articulation test, no child showed alterations in phonemes / a / b / e / f / g / i / j / k / m / o / p /. The most frequently altered phonemes were / d / t / s / ch / ñ /. Distortion was the most frequent alteration in the three types of AOB, and the most altered phonemes were / d /in 62.9 %, and / t / in 51.5 % (Table 2).

**Table 2:** Frequency of distortion by tongue thrust and tongue interposition in children with AOB

Consonant	Distortion by tongue thrust	Distortion by tongue interposition	Normal
D /	7 (5.3 %)	76 (57.6 %)	49 (37.1 %)
T /	6 (4.5 %)	62 (47 %)	64 (48.5 %)
S /	21 (15.9 %)	33 (25 %)	78 (59.1 %)
ch /	3 (2.3 %)	13 (9.8 %)	116 (87.9 %)
ñ /	1 (0.8 %)	12 (9.1 %)	119 (90.2 %)

No significant association between different types of dyslalias with mild, moderate and severe AOB (*p*-value = 0.974) (Table 3) was found. No differences between gender and type of dyslalia, praxias, oral structures, or AOB magnitude were found. Table 3 describes the type of dyslalia, praxias, and intraoral structures regarding AOB magnitude.

**Table 3:** Relation between the magnitude of anterior open bite (AOB) and the type of dyslalia, praxia, and intraoral structures

Variable	AOB magnitude, *n* (%)			
	Mild	Moderate	Severe	
Type of dyslalia				Total
Distortion	28 (28 %)	65 (65 %)	7 (7.0 %)	100
Omission	0 (0.0 %)	0 (0.0 %)	0 (0.0 %)	0
Substitution	0 (0.0 %)	1 (100 %)	0 (0.0 %)	1
Distortion and replacement	0 (0.0 %)	1 (100 %)	0 (0.0 %)	1
No dyslalia	7 (23.3 %)	21 (70 %)	2 (6.7 %)	30
Movement of lips right and left together				
Accomplished	29 (24.6 %)	80 (67.8 %)	9 (7.6 %)	118
Not accomplished	6 (42.9 %)	8 (57.1 %)	0 (0.0 %)	14
Palatal rugae				
Normal	20 (23.8 %)	59 (70.2 %)	5 (6.0 %)	84
Thick	8 (25 %)	21 (65.6 %)	3 (9.4 %)	32
Thin	7 (43.8 %)	8 (50 %)	1 (6.3 %)	16
Palatal sensitivity				
Normal	22 (23.4 %)	65 (69.1 %)	7 (7.4 %)	94
Reduced	11 (33.3 %)	20 (60.6 %)	2 (6.1)	33
Increased	2 (40 %)	3 (60 %)	0 (0.0 %)	5

## Discussion

The results showed a low prevalence of AOB in the studied population. 77.4 % exhibited speech disorders. These results are consistent with the findings of Khinda *et al.* [15], Mehnert [7], Alvarez *et al.* [5], and other authors cited in the review by Johnson *et al.* [6], who indicated that disorders in phonemes are strongly associated with AOB. However, studies showed that AOB was not only related to phonation problems, but to other types of malocclusions. However, Farronato *et al.* [16], Laine [17], Pahkala *et al.* [18], or studies cited by Johnson *et al.* (Hopkin and McEwen in 1956) [6] stated that phonetic problems include people with malocclusion and those with normal occlusion. In the present study, no speech evaluation was performed on children with normal occlusion.

According to the results of the present investigation, 75.8 % of patients who presented AOB showed distortion dyslalia. This finding is in disagreement with Alvarez *et al.* [5], who reported that patients with open bite exhibited similar percentages of omission, distortion, and replacement of phonemes. However, this difference might be explained by differences in geographical location among groups because, even though Spanish is spoken, accents and pronunciation are different. This fact highlights the importance of assessing the frequency of dyslalias in children with AOB in each region to conduct a timely and targeted therapy. Furthermore, discrepancies in the results of Alvarez *et al.* might also be explained by the age of the patients as they were different from this study.

The greatest speech difficulty was represented by dental articulation phonemes, as it was reported by Fymbo (cited by Johnson *et al.* [6]), who found that patients with malocclusion showed greater difficulty with the sounds of dental pronunciation.

The phonemes / t / d / have a dental place of articulation; / s / is alveolar, and / ch / ñ / are palatal. No point of articulation forces the tongue to protrude between the dental arches. Nonetheless, one of the findings observed in this study was that phonetic distortion of these phonemes modifies tongue position, causing interdental tongue thrust, which may lead to perpetuation and recurrence of AOB after correction achieved by orthodontic treatment.

The results of the present investigation are in agreement with the findings of Sahad *et al.* [19], who showed that there is a significant relationship between AOB and hissing. Similarly, Pomerantz and Zeller, cited by Johnson *et al.* [6], concluded that open bite is significantly related to the sound of the phonemes / s / z / d / l /. However, the age of the patients evaluated in that investigation differs from the patients assessed in the present study.

Farronato *et al.* [16] concluded that the effect of occlusion in dyslalias seems to increase proportionally as the severity of the malocclusion increases. This is in disagreement with the present study, where a significant association between mild, moderate, and severe AOB with various dyslalia types was not found. The most frequent dyslalia found in that work was distortion (75.8 %). In relation to AOB magnitude, it was found that 65 patients (49.2 %) showed moderate AOB. Rathbone [20] and Doshi *et al.* [4] concluded that there is a relationship between malocclusion and speech problems, but a correlation with the severity of this condition was not established. Johnson *et al.* [6], after extensive reviewing, concluded that certain dental irregularities show relation to phonation disorders, but this does not seem to be related to the severity of the malocclusion. Conclusive evidence to suggest that alterations in teeth position may improve speech disorders is yet to be found. This is consistent with the findings of the present study.

It is important to point that performing some praxias, including movement of the lips together to right and left, becomes difficult in patients with AOB due to labial incompetence and hypotonicity of the surrounding tissues. Only one patient, who was not capable of sweeping the tongue against the palate, presented problems of coordination with the phonemes / l / n / due to a pulling lingual frenulum, which hindered the proper tongue positioning during phonation. This incidental finding was not considered as an exclusion criterion in the present study. An important aspect of mobility and lingual functionality is the lingual frenulum, which is assessed through direct observation by looking at the insertion and length of the lingual frenulum and its mobility considering the vertical, sagittal, lateral, and diagonal planes [13].

## Conclusion

Based on the results of this study, it may be emphasized that occlusal problems are related to phonetic problems. However, further investigation is required to elucidate whether AOB leads to phonetic changes or phonetic alterations cause AOB. For this purpose, cohort studies are required to early diagnose and manage AOB and language disorders. Since no studies were found that compared the relationship between AOB and praxias, this study provides valuable information for future research and suggests exhaustive evaluation of muscle tone for timely referral to the phonoaudiologist.

The main strengths of this study were the interdisciplinary work between the areas of orthodontics and phonoaudiology, the adaptation of phonemes tables to a Colombian population to eliminate potential confounding variables, and the sample size was representative of the population for the ages studied. The fact that this sample was representative was a major asset because public educational and corrective programs may be introduced for this population.

Two limitations of this study were not considered as exclusion criteria: the insertion of the lingual frenulum and the presence of hearing problems. The presence of a short or pulling lingual frenulum may distort phonemes; however, only one case was found in a child with AOB. Hearing problems was not assessed in the present investigation, although they may alter the proper pronunciation of phonemes.

Finally, the prevalence of AOB in Envigado is low (2.7 %), whereas phonation alterations are very common (77.8 %), with distortion dyslalia being the most frequently found (75.8 %).

## Abbreviation

AOB: Anterior open bite
